# *Cynomorium songaricum* extract enhances novel object recognition, cell proliferation and neuroblast differentiation in the mice via improving hippocampal environment

**DOI:** 10.1186/1472-6882-14-5

**Published:** 2014-01-07

**Authors:** Dae Young Yoo, Jung Hoon Choi, Woosuk Kim, Hyo Young Jung, Sung Min Nam, Jong Whi Kim, Yeo Sung Yoon, Ki-Yeon Yoo, Moo-Ho Won, In Koo Hwang

**Affiliations:** 1 Department of Anatomy and Cell Biology, College of Veterinary Medicine, and Research Institute for Veterinary Science, Seoul National University, Seoul 151-742, South Korea; 2 Department of Anatomy, College of Veterinary Medicine, Kangwon National University, Chuncheon 200-701, South Korea; 3 Department of Oral Anatomy, College of Dentistry, Research Institute of Oral Sciences, Gangneung-Wonju National University, Gangneung 210-702, South Korea; 4 Department of Neurobiology, School of Medicine, Kangwon National University, Chuncheon 200-701, South Korea

**Keywords:** *Cynomorium songaricum* extract, Hippocampus, 5-bromodeoxyuridine, Polysialylated neural cell adhesion molecule, Corticosterone

## Abstract

**Background:**

*Cynomorium songaricum* Rupr. (CS) has been used as a medicine to treat many diseases as well as to alleviate age-related issues, such as memory impairment, dementia, and stress. In this study, we assessed the effects of *Cynomorium songaricum* extract (CSE) on the novel object recognition, cell proliferation and neuroblast differentiation in the dentate gyrus of mice by using 5-bromodeoxyuridine (BrdU) and polysialylated neural cell adhesion molecule (PSA-NCAM). We also measured serum corticosterone levels to assess its correlation with neurogenesis and stress.

**Methods:**

Male C57BL/6 J mice were divided into 3 groups: vehicle-treated, 40 mg/kg CSE-treated, and 100 mg/kg CSE-treated. The vehicle and CSE were given to mice once a day for 3 weeks. BrdU was injected twice a day for 3 days to label newly generated cells.

**Results:**

Administration of CSE significantly increased the preferential exploration of new objects in these mice. In addition, administration of CSE decreased serum levels of corticosterone. BrdU-positive cells as well as brain-derived neurotrophic factor (BDNF) mRNA expression in the dentate gyrus were higher in the CSE-treated groups than in the vehicle-treated group. PSA-NCAM-positive neuroblasts and their well-developed tertiary dendrites were also significantly increased by the treatment of CSE. These effects were prominent at the higher dosage than at the lower dosage.

**Conclusion:**

These results suggest that administration of CSE increases cell proliferation and neuroblast differentiation in the dentate gyrus of mice by reducing serum corticosterone levels and increasing BDNF levels in this area.

## Background

Findings of recent research suggest that neurogenesis occurs throughout the lifetime in the adult mammalian brain [1-4]. There are 2 discrete neurogenic regions in the adult brain; the dentate gyrus is one of them [5]. The dentate gyrus is closely related to learning and memory [6,7], and it has been reported that hippocampal neurogenesis is associated with neurological problems, such as depression and Alzheimer’s disease [8,9]. In the dentate gyrus, new neurons are produced from progenitor cells in the subgranular zone and migrate into the granule cell layer [2]. Neurogenesis in the adult dentate gyrus is highly labile [10] and is affected by many exogenous and endogenous factors [11,12].

*Cynomorium songaricum* Rupr. (CS) is a well-known root-parasitic plant and is distributed throughout northwest China [13]. *Cynomorium songaricum* extract (CSE) has been widely used as Chinese herbal remedy for problems such as sexual dysfunction, renal disease, and lumbar weakness [14]. Moreover, it has been reported that CS induces proliferation of undifferentiated spermatogonia with glial cell-derived neurotrophic factor (GDNF) enhancement [15]. Chemically, CS consists of organic acids, triterpenes, polysaccharides, steroidal compounds, and flavonoids [16]. CSE has been shown to suppress age-related learning impairments in aged flies by reducing hydroperoxide levels and increasing antioxidants [14]. Among the chemical compounds in CS, flavonoids have antioxidant effects, and certain types of flavonoids are involved in neuroprotection and hippocampal neurogenesis [17-19]. It has also been reported that CSE modulates γ-aminobutyric acid (GABA) and monoamine transporters, both of which are involved in hippocampal neurogenesis [13].

Novel object recognition tasks are non-aversive learning paradigms, which rely on spontaneous exploratory behavior in experimental animals. Therefore, we investigated the effects of CSE on novel object recognition and hippocampal neurogenesis by using 5-bromodeoxyuridine (BrdU) for cell proliferation and polysialylated neural cell adhesion molecule (PSA-NCAM) for detecting developing granule neurons in mice. We also measured serum corticosterone and brain-derived neurotrophic factor (BDNF) mRNA expression levels to elucidate the possible effect of CSE on neurogenesis.

## Methods

### Preparation of CSE

*Cynomorium songaricum* Rupr. was purchased from Kyung-dong market (Seoul, South Korea). The plants were authenticated by two oriental medical doctors and the voucher specimen was deposited in our laboratory (deposition number: 2012–028). The plants (100 g) were chopped and blended using a blender and soaked in 2 L of 80% ethanol and refluxed at 20°C for 2 h three times. The insoluble materials were removed through centrifugation at 10,000 × *g* for 30 min, and the resulting supernatant was concentrated and freeze-dried to yield a powder (yield: 13.2%). Before each experiment, the dried extract was dissolved in distilled and deionized water.

### Experimental animals

The progeny of male C57BL/6 J mice were purchased from the Jackson Laboratory Co. Ltd (Bar Harbor, ME). Six-week-old mice were used in this study. The mice were housed in a conventional state under adequate controlled temperature (22°C), humidity (55%), 12-h light/ 12-h dark cycle, and provided with free access to food and tap water. The procedures for the care and handling of animals conformed to guidelines that are in compliance with current international laws and policies (NIH Guide for the Care and Use of Laboratory Animals, NIH Publication No. 85–23, 1985, revised 1996), and the experimental protocol was approved by the Institutional Animal Care and Use Committee (IACUC) of Seoul National University (approval no. SNU-110527-2). All of the experiments were conducted in a way as to minimize the number of animals used and the suffering caused by the procedures used in the present study.

### Treatment with CSE and BrdU

Mice were randomly divided into the 3 groups: vehicle-treated group, 40 and 100 mg/kg CSE-treated groups (*n* = 12 in each group). Vehicle or CSE were orally administered to mice using a feeding needle once a day for 21 days and the animals were sacrificed at immediately after the novel object recognition test. These schedules were adopted because PSA-NCAM is a marker for differentiated neuroblasts expressed in immature neurons from only 1 to 21 days of cell age. In order to determine the integration of new neurons generated in the adult brain, the animals in the vehicle-treated group (*n* = 7) and the 40/100 mg/kg CSE-treated groups (*n* = 7 in each group) were treated with 50 mg/kg 5-bromodeoxyuridine (BrdU, Sigma, St. Louis, MO) twice a day for 3 days from the day of the first CSE treatment.

### Novel object recognition test

**Figure 1:**
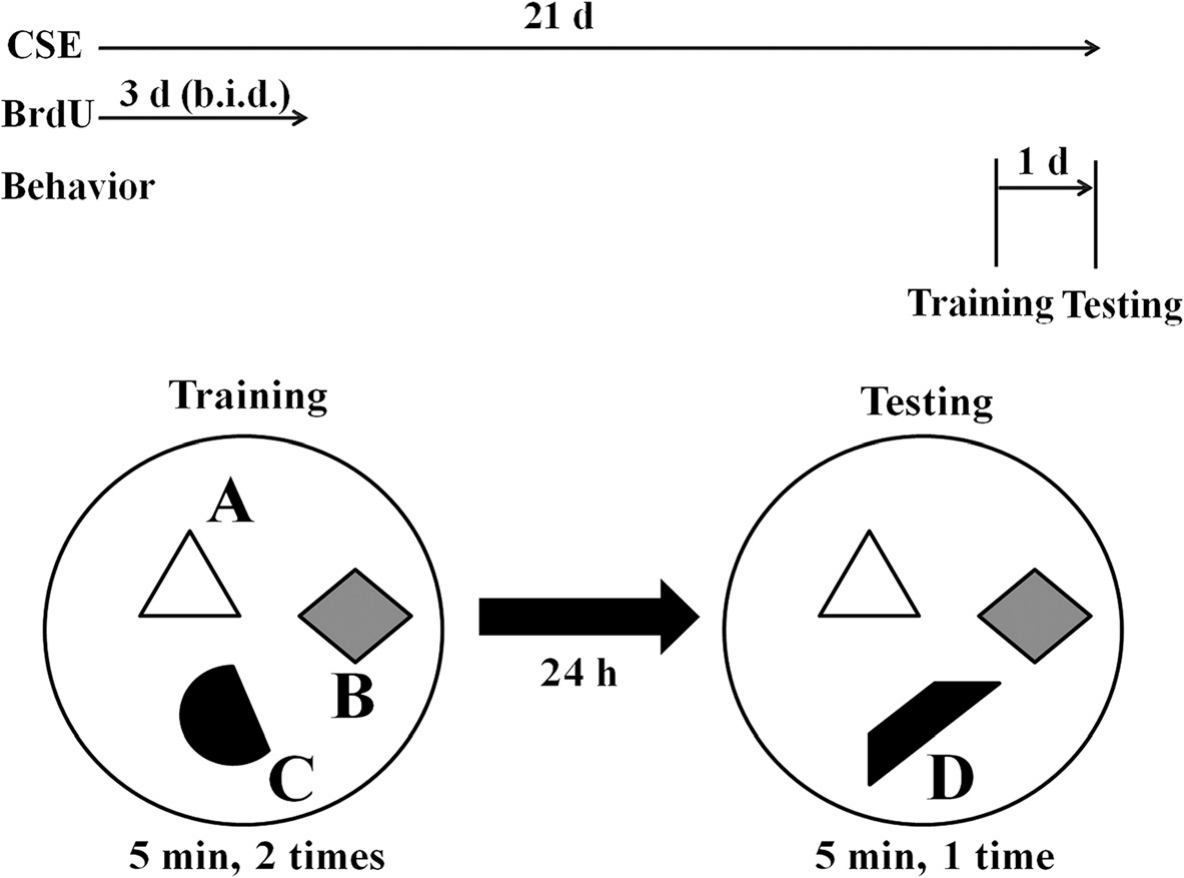
**Study design and novel object recognition task.** Vehicle or CSE were orally administered to mice using a feeding needle once a day for 21 days and BrdU was treated twice a day for 3 days from the day of the first CSE treatment. At 20 days after CSE treatment, mice were allowed to explore three different objects (A, B and C) for 5 min trials in an open field. 24 h after open field trial, novel object recognition test was performed and exploration times were recorded for each object.

At 20 days after CSE treatment, mice (*n* = 12 in each group) were placed in an open field and allowed to explore three different objects (A, B and C) for 5 min trials and this was repeated once. Object C was replaced by a novel object (D) 24 h after open field trial and the mice were allowed to explore for 5 min trial (Figure 1). Exploration times were recorded for each object and expressed as a percentage of total exploration time.

### Blood sampling and measurement of corticosterone levels

For blood sampling, the animals (*n* = 12 in each group) were anesthetized with intraperitoneal injection of 1 g/kg urethane (Sigma, St. Louis, MO), and blood samples were collected from each mouse by cardiac puncture before tissue sampling for immunohistochemistry and PCR analysis. Serum was separated from the blood by centrifugation at 1,100 g for 15 min at 4°C and serum (50 μL) was added to 5 mL of methylene chloride and incubated at room temperature for 10 min. After filtration with cheesecloth, the mixture was combined with 2.5 mL of fluorescence reagent (7:3, sulfuric acid/absolute ethanol), vortexed vigorously and incubated for 30 min at room temperature. After centrifugation, the lower layer was measured using a spectrophotometer (excited wavelength, 475 nm; emission wavelength, 530 nm).

### Tissue processing for histology

For histology, the animals (*n* = 7 in each group) were used after blood sampling. The animals were perfused transcardially with 0.1 M phosphate-buffered saline (PBS, pH 7.4) followed by 4% paraformaldehyde in 0.1 M phosphate-buffer. The brains were removed and postfixed in the same fixative for 4 h. The brain tissues were cryoprotected by infiltration with 30% sucrose overnight. Thirty-μm-thick brain sections in coronal plane were serially cut using a cryostat (Leica, Wetzlar, Germany). The sections were collected into six-well plates containing PBS for further process.

### Immunohistochemistry for BrdU and PSA-NCAM

To obtain the accurate data for immunohistochemistry, the free-floating sections were carefully processed under the same conditions. The tissue sections were selected between −1.46 mm and −2.46 mm posterior to the bregma in reference to the mouse atlas for each animal [20]. The sections were sequentially treated with 0.3% hydrogen peroxide (H_2_O_2_) in PBS for 30 min and 10% normal goat serum in 0.05 M PBS for 30 min. For BrdU immunostaining, DNA was first denatured by incubating the sections in 50% formamide/2× standard saline citrate at 65°C for 2 h and in 2 N HCL at 37°C for 30 min. They were next incubated with diluted mouse anti-PSA-NCAM (1:200; Chemicon, Temecula, CA) or rat anti-BrdU (1:200, BioSource International, Camarillo, CA) overnight at room temperature and subsequently exposed to biotinylated goat anti-mouse IgG (1:200; Vector, Burlingame, CA) or FITC-conjugated anti-rat IgG (1:200; Jackson ImmunoResearch, West Grove, PA). For PSA-NCAM staining, the sections were incubated with streptavidin peroxidase complex (1:200; Vector), visualized with reaction to 3,3’-diaminobenzidinetetrachloride (Sigma, St. Louis, MO) in 0.1 M Tris–HCl buffer (pH 7.2) and mounted on gelatin-coated slides.

In order to quantitatively analyze BrdU- and PSA-NCAM-positive cells, 10 sections per animal selected in the hippocampus at the level of −1.46 to −2.46 mm posterior to the Bregma. BrdU- and PSA-NCAM-positive cells were calculated using an image analyzing system equipped with a computer-based CCD camera (software: Optimas 6.5, CyberMetrics, Scottsdale, AZ). The cell counts from all of the sections of all of the mice were averaged.

### BDNF mRNA levels in the dentate gyrus

For PCR analysis, the animals (*n* = 5 in each group) were used in this study after blood sampling. The dentate gyrus was dissected out from brain tissues. RNA extraction was performed using a total RNA isolation kit (Macherney-Nagel). Spectrophotometric measurements were conducted using the Nanodrop Spectrophotometer (Nanodrop Technologies Wilmington, DE, USA) to determine RNA concentration and purity. A high capacity cDNA archive kit (Applied Biosystems) was used to reverse transcribe the equalized RNA samples. Quantitative real-time PCR (qPCR) was performed with 50 ng cDNA using custom-designed gene-expression assays for BDNF.

Gene expression of targets was assessed using Taqman gene expression assays (Applied Biosystems, UK) containing specific target primers, and FAM-labeled MGB target probes. β-actin gene expression was used to normalize gene expression between samples, and was quantified using a β-actin endogenous control gene expression assay containing specific primers, and a VIC-labeled MGB probe for rat β-actin. Analysis was performed using the ΔΔCT method. The primers used were as follows: 5′-GTGACAGTATTAGCGAGTGGG −3′ (forward) and 5′-CTAGGGCGGCCCACGATGGA-3′ (reverse) for BDNF and 5’-GCACCACACCTTCTA CAATG-3′ (forward) and 5′-TGCTTGCTGATCCACATCTG-3′ (reverse) for β-actin.

### Statistical analysis

The data shown here represent the means of experiments performed for each experimental area. Differences among the means were statistically analyzed by one-way analysis of variance followed by Tukey’s multiple range method in order to elucidate differences between the vehicle- and CSE-treated groups.

## Results

### Effects of CSE on object recognition

**Figure 2:**
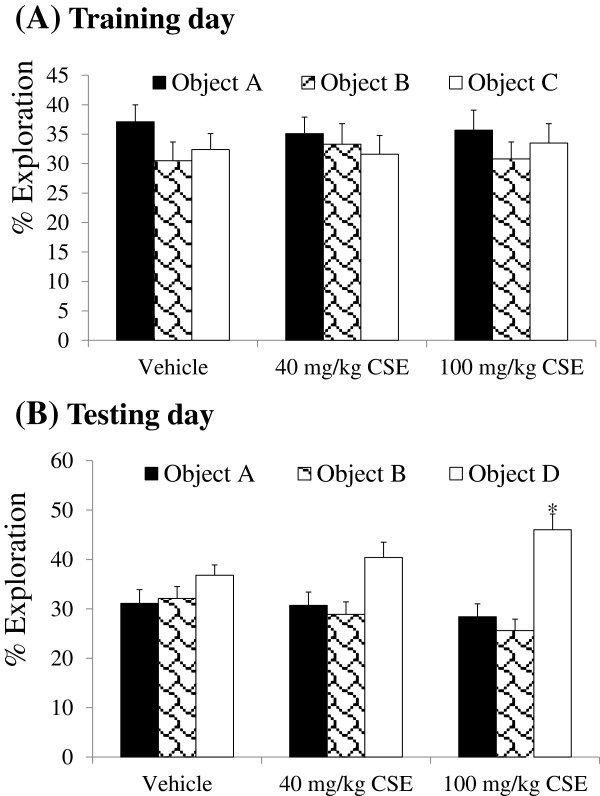
**Effects of CSE on novel object recognition test.** Preference for exploring novel objects in vehicle-treated, 40 mg/kg CSE-treated, and 100 mg/kg CSE-treated mice (*n* = 12 per group; *P < 0.05, versus vehicle-treated group). Data are presented as the exploration time for each object (A, B, or C, where C is replaced by D on the testing day) as a percentage of the total exploration time. On the training day, the animals do not show preference to explore any particular object. On the testing day, CSE-treated mice preferentially explore the novel object D. All data are shown as % exploration ± SEM.

During the training period, all groups spent a similar fraction of time exploring the 3 different objects. In addition, there were no significant changes in total exploration time. During the testing period, vehicle-treated mice showed significant increases in the amount of time spent exploring object D. The administration of CSE significantly increased the time spent exploring object D in a dose-dependent manner and the time spent exploring object D in 100 mg/kg CSE-treated group was significantly increased compared to that in the vehicle-treated group (Figure 2).

### Effects of CSE on serum corticosterone levels

**Figure 3:**
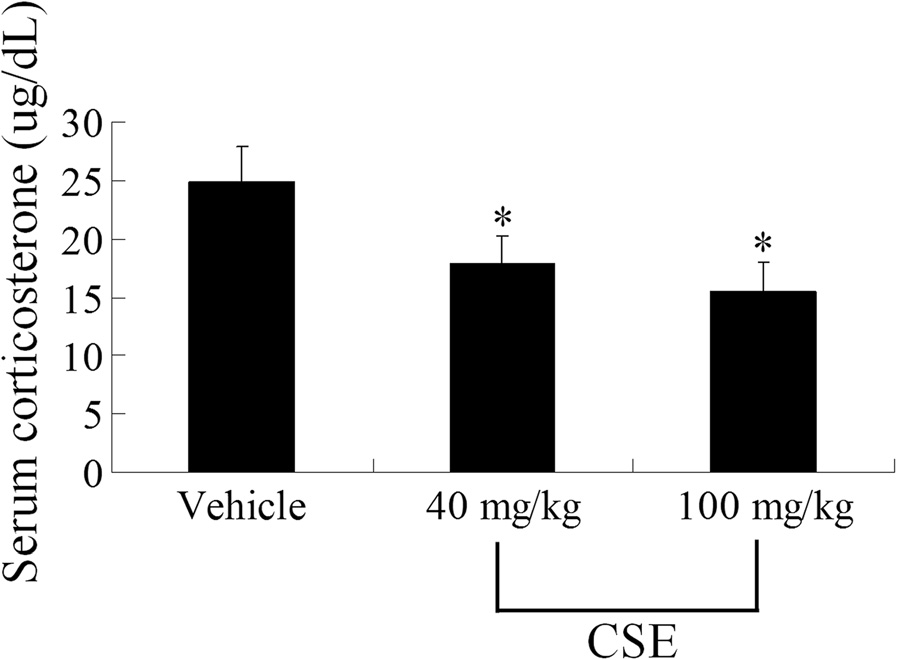
**Effects of CSE on serum corticosterone levels.** Serum corticosterone levels in the vehicle-treated, 40 mg/kg CSE-treated, and 100 mg/kg CSE-treated groups (*n* = 12 per group; *P < 0.05, versus vehicle-treated group). All data are shown as the mean ± SEM.

To elucidate the possible mechanism of CSE action on neurogenesis, serum corticosterone levels were measured in each group. In the vehicle-treated group, mean serum corticosterone was 24.8 μg/dL. In the 40 mg/kg CSE-treated group, mean serum corticosterone level was 17.8 μg/dL, which was significantly lower than that in the vehicle-treated group. In the 100 mg/kg CSE-treated group, mean serum corticosterone level was 15.4 μg/dL, which indicated that the reduction was dose-dependent (Figure 3).

### Effects of CSE on cell proliferation

**Figure 4:**
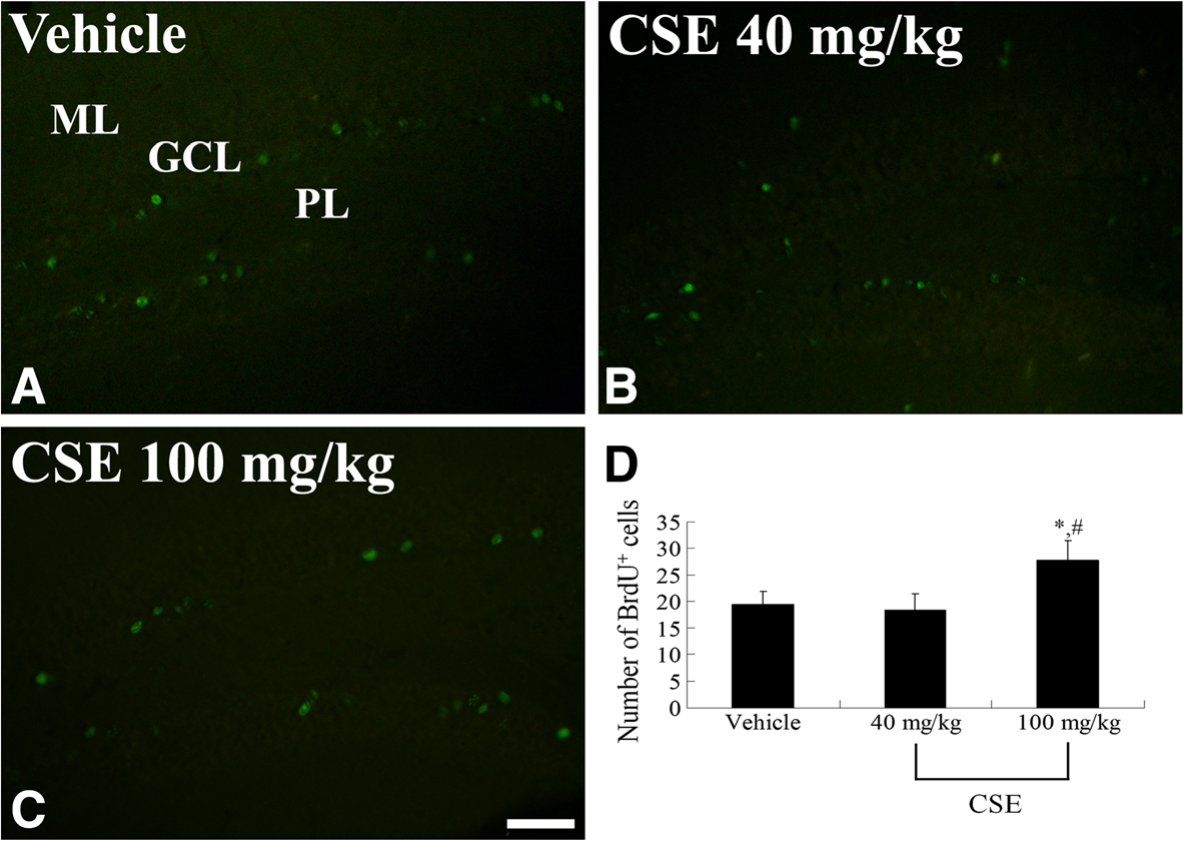
**Effects of CSE on cell proliferation.** BrdU immunofluorescence in the dentate gyrus of the vehicle-treated **(A)**, 40 mg/kg CSE-treated **(B)**, and 100 mg/kg CSE-treated **(C)** groups. BrdU-positive cells are significantly higher in the 100 mg/kg CSE-treated group than in the vehicle-treated and 40 mg/kg CSE-treated groups. GCL: granule cell layer; ML: molecular layer; PoL: polymorphic layer. Scale bar = 50 μm. **D**: The number of BrdU-positive cells in the dentate gyrus of the vehicle-treated, 40 mg/kg CSE-treated, and 100 mg/kg CSE-treated groups (*n* = 7 per group; *P < 0.05, versus vehicle-treated group; ^#^*P* < 0.05, versus the 40 mg/kg CSE-treated group). All data are shown as the mean ± SEM.

Immunohistochemical staining for BrdU was performed to confirm the effect of CSE on cell proliferation in the dentate gyrus (Figure 4). In the vehicle group, a few BrdU-positive cells were detected in the dentate gyrus (Figure 4A). Compared to the vehicle-treated group, the 40 mg/kg CSE-treated group showed little difference in the number of BrdU-positive cells (Figure 4B). However, the number of BrdU-positive cells in the dentate gyrus was significantly higher in the 100 mg/kg CSE-treated group than in the vehicle-treated and 40 mg/kg CSE-treated groups (Figure 4C and D).

### Effects of CSE on neuroblast differentiation

**Figure 5:**
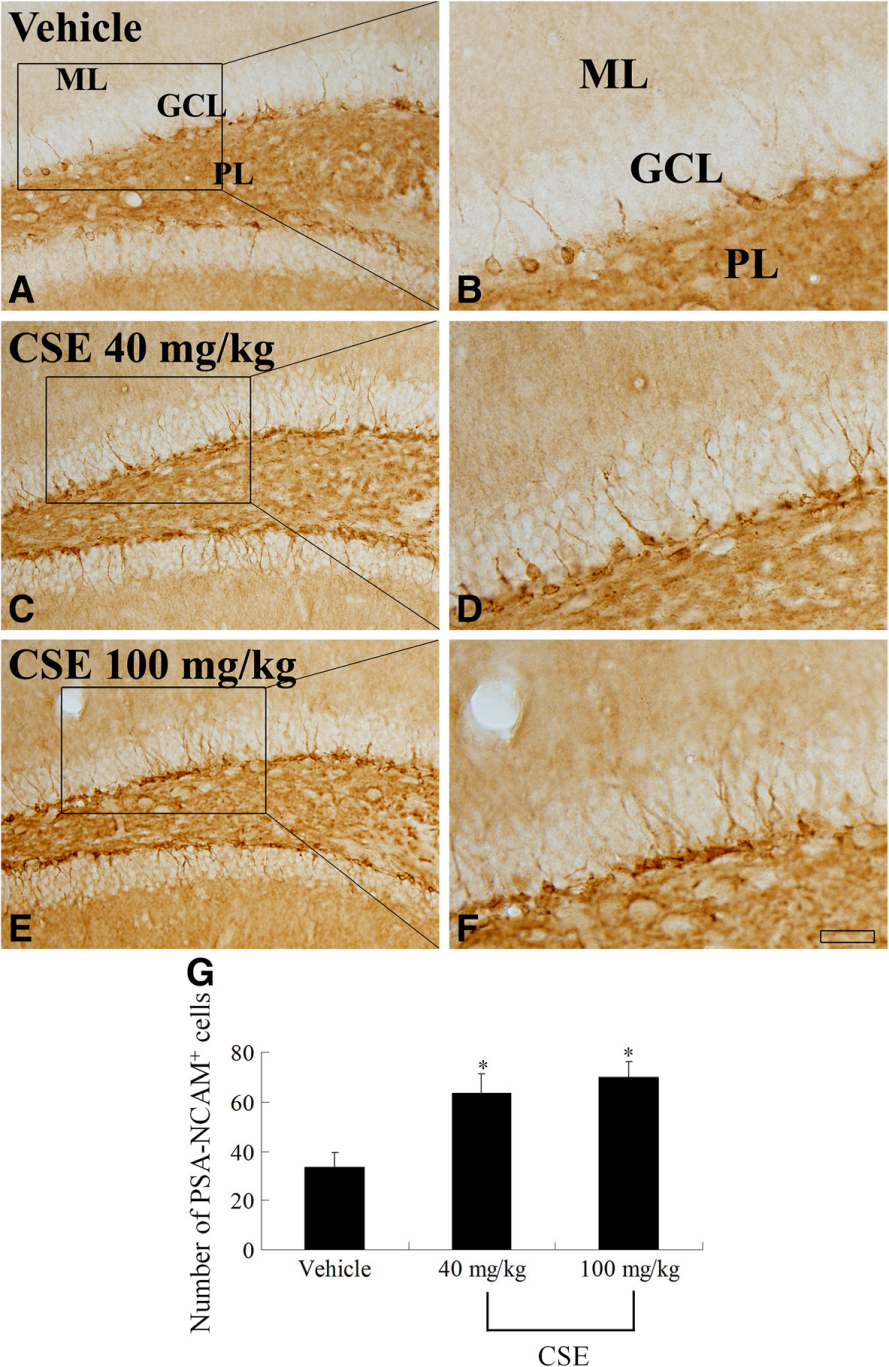
**Effects of CSE on neuroblast differentiation.** PSA-NCAM immunohistochemistry in the dentate gyrus of the vehicle-treated **(A, B)**, 40 mg/kg CSE-treated **(C, D)**, and 100 mg/kg **(E, F)** CSE-treated groups. Compared with the vehicle-treated group, the CSE-treated group shows a higher number of PSA-NCAM-immunoreactive neuroblasts with well-developed dendrites. GCL: granule cell layer; ML: molecular layer; PoL: polymorphic layer. Scale bar = 50 μm **(A, C, and E)**, 25 μm **(B, D, and F)**. **G**: The number of PSA-NCAM-positive neuroblasts in the dentate gyrus of the vehicle-treated, 40 mg/kg CSE-treated, and 100 mg/kg CSE-treated groups (*n* = 7 per group; *P < 0.05, versus vehicle-treated group). All data are shown as the mean ± SEM.

To observe neuroblast differentiation in the subgranular zone of mouse dentate gyrus, we performed immunohistochemical staining using antibody against PSA-NCAM (Figure 5). In the vehicle-treated group, PSA-NCAM-positive neuroblasts with poorly developed dendrites were noted in the subgranular zone (Figure 5A and B). The number of PSA-NCAM-positive neuroblasts was significantly higher in the 40 and 100 mg/kg CSE-treated groups than in the vehicle-treated group. Moreover, these neuroblasts had well-developed dendrites extending to the molecular layer (Figure 5C, D, E, F, and G).

### Effects of CSE on BDNF mRNA expression

**Figure 6:**
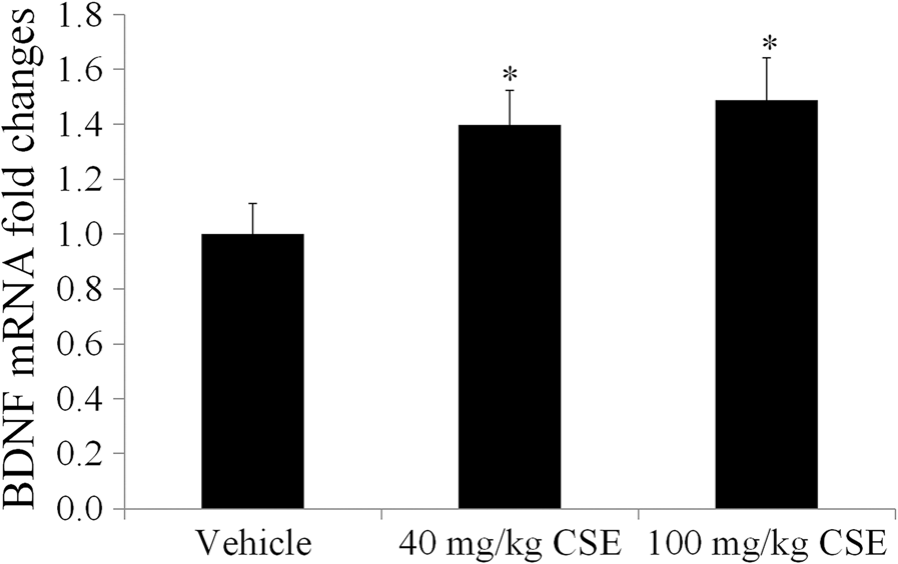
**Effects of CSE on BDNF mRNA expression.** BDNF mRNA expression in the dentate gyrus of the vehicle-treated, 40 mg/kg CSE-treated, and 100 mg/kg CSE-treated groups as assessed by quantitative PCR (*n* = 5 per group; *P < 0.05, versus vehicle-treated group). BDNF mRNA expression in the dentate gyrus is higher in the CSE-treated groups than in the vehicle-treated group. All data are shown as the ratio to vehicle-treated group ± SEM.

To investigate the possible mechanisms underlying the observed cognitive improvements, we tested the dentate gyri of mice for BDNF expression. BDNF mRNA expression in the 40 and 100 mg/kg CSE-treated groups was significantly increased by 1.40 and 1.49 folds compared to that in the vehicle-treated group, respectively (Figure 6).

## Discussion

In the present study, we observed the behavioral preferences of mice to novel objects after CSE treatment; this behavioral paradigm exploits the ability of the mice to explore novel objects over familiar ones during simultaneous presentation and has been employed to evaluate recognition memory [21]. During training periods, mice in all groups did not show any preferences among the 3 different objects. However, during the test periods, CSE-treated mice showed strong preferences for the novel object, while vehicle-treated mice displayed a much lesser preference for it. The result of the behavioral test is supported by changes of serum corticosterone and BDNF expression measured in this study.

We checked serum corticosterone and BDNF mRNA levels to elucidate the possible mechanisms for hippocampal neurogenesis. CSE treatment decreased serum corticosterone levels in general, and the corticosterone level in the 100 mg/kg CSE-treated group was lower than that in the 40 mg/kg CSE-treated group. It has been reported that corticosteroid hormones regulate the proliferation, survival, and death of granule cells in the dentate gyrus [22-24]. Additionally, corticosterone is known to be associated with cognitive impairment and has detrimental effects on LTP in the dentate gyrus [25-27]. Moreover, corticosterone acts on hippocampal BDNF expression through the exon IV promoter [28], which is required for the enhancement of hippocampal neurogenesis [29].

Next, we morphologically observed the effects of CSE on cell proliferation and neuroblast differentiation in the dentate gyrus. Treatment with CSE increased both BrdU-positive cells and PSA-NCAM-positive neuroblasts. These neuroblasts displayed well-developed dendrites, indicating that CSE may be involved in neuroblast maturation. Therefore, treatment with CSE can possibly modulate synaptic plasticity by increasing cell proliferation and neuroblast differentiation in the subgranular zone of the dentate gyrus. It has been reported that functional and morphological changes in the synapse induce altered neuronal plasticity in the mammalian brain [30,31]. Additionally, CSE is known to act as an inhibitor of serotonin and GABA transporters, both of which are crucial for limiting monoaminergic and GABAergic activity [13,32,33]. GABA is the principal inhibitory neurotransmitter in the brain and plays an important role in CNS development and plasticity [34], and serotonin is an important modulator of hippocampal neurogenesis [35]. GABA also significantly increases recognition and working memory in rats [36].

Administration of CSE significantly increased BDNF mRNA expression in the dentate gyrus homogenates. BDNF, synthesized locally in the dendrites of granule cells, promotes differentiation and maturation of progenitor cells in the subgranular zone of dentate gyrus by enhancing GABA release [37]. Here, the increase of tertiary dendrites of the neuroblasts, stained with PSA-NCAM, may be associated with BDNF increase in the dentate gyrus.

Recently, many researchers have explored the mechanism of action and function of CSE in the brain. CSE has been shown to attenuate Aβ-induced cell death and protect against superoxide anion-induced cell death [38,39]. In addition, CSE exhibits anti-aging effects by increasing telomere length in the brain of the D-galactose-induced aging mice [40]. In this study, we confirmed that CSE administration influences hippocampal neurogenesis and cognitive memory by changing BDNF and serum corticosterone levels.

## Conclusion

Treatment with CSE significantly increased preference for the novel object in the novel object recognition test and enhanced hippocampal neurogenesis in the mouse dentate gyrus. Changes of the BDNF and serum corticosterone levels were also detected and may be closely associated with novel object recognition and hippocampal neurogenesis. These results suggest that CSE has the potential for enhancing hippocampal plasticity and can be used to people who have problems in hippocampal memory.

## Competing interests

The authors declare that they have no competing interests.

## Authors’ contributions

DYY, JHC, WK, HYJ, SMN, JWK, YSY, and IKH conceived the study, designed and conducted the experiments, and drafted the manuscript. KYY and MHW participated in designing and discussing the study. All authors have read and approved the final manuscript.

## Pre-publication history

The pre-publication history for this paper can be accessed here:

http://www.biomedcentral.com/1472-6882/14/5/prepub
